# The Use of Recycled Poly(Ethylene Terephthalate)/Amorphous Polyester Blends/Composites in Materials Extrusion (MEX) Additive Manufacturing Techniques: The Influence of Talc and Carbon Fiber on the Mechanical Performance and Hear Resistance

**DOI:** 10.3390/polym18060768

**Published:** 2026-03-22

**Authors:** Jacek Andrzejewski, Natan Zelewski, Wiktoria Gosławska, Adam Piasecki, Patryk Mietliński, Frederik Desplentere, Aleksander Hejna

**Affiliations:** 1 Institute of Materials Technology, Poznan University of Technology, Piotrowo 3 Street, 61-138 Poznan, Poland; aleksander.hejna@put.poznan.pl; 2 Faculty of Mechanical Engineering, Poznan University of Technology, Piotrowo 3 Street, 61-138 Poznan, Poland; natan.zelewski@student.put.poznan.pl; 3 Faculty of Materials Engineering and Technical Physics, Poznan University of Technology, Piotrowo 3 Street, 61-138 Poznan, Poland; wiktoria.goslawska@student.put.poznan.pl; 4 Institute of Materials Science and Engineering, Poznan University of Technology, Piotrowo 3 Street, 61-138 Poznan, Poland; adam.piasecki@put.poznan.pl; 5 Institute of Mechanical Technology, Poznan University of Technology, Piotrowo 3 Street, 61-138 Poznan, Poland; patryk.mietlinski@put.poznan.pl; 6 Research Group ProPoliS, Department of Materials Engineering, KU Leuven Campus Bruges, Spoorwegstraat 12, St.-Michiels, B-8200 Brugge, Belgium; frederik.desplentere@kuleuven.be

**Keywords:** polymer blends, poly(ethylene terephthalate), extrusion 3D printing (MEX), recycling, polymer composite, thermal properties, talc, carbon fibers

## Abstract

The conducted study was focused on the development of a new type of polymer blends intended for additive manufacturing applications, in particular, the material extrusion method (MEX). The developed materials were prepared from recycled poly(ethylene terephthalate) and amorphous copolymers poly(ethylene terephthalate-glycol) (PETG), and poly(cyclohexylenedimethyl terephthalate-glycol) (PCTG). The basic blend systems were additionally modified with POE-g-GMA impact modifier (IM) during the reactive extrusion process. The main aim of the work was to assess the effectiveness of using composite additives and their influence on the mechanical and thermomechanical parameters of the tested systems. To prepare the composites, selected polymer blends were modified with 10% of talc (T) and carbon fibers (CF). The properties evaluation includes the mechanical/thermomechanical testing, thermal analysis and structural observations. The accuracy of printing was measured using optical scanning methods. The test results indicate that even the relatively small amount of the CF filler could lead to a significant increase in tensile modulus from reference 1.6 GPa to 2.9 GPa; the same improvement applies to strength values, where the CF-modified materials reached 45 MPa, compared to the reference 31 MPa. The heat deflection tests (0.455 MPa) after annealing revealed the maximum HDT of around 170 °C for both types of CF-modified materials. The Vicat test results were also favorable for annealed materials. Considering that the Vicat/HDT results after the 3D-printing process usually reach around 70 °C, the performed heat treatment strongly enhanced the heat resistance for most of the prepared blends. The performed studies revealed that for most of the prepared materials, the brittleness was a common drawback for both MEX-printed and injection-molded materials.

## 1. Introduction

The development of modern polymer processing techniques cannot be discussed without the additive manufacturing methods (AM). The new processing methods or new materials developed for the AM processing are introduced to the market, even a few times a year [1,2,3,4,5,6,7,8]. In comparison, for the traditional polymer processing techniques, like injection molding, the development and implementation time is much slower [9,10,11]. This is mostly caused by differences in the complexity of the standard and AM-based technologies, as well as the cost of machine/tool manufacturing. The most dynamic development concerns the popular manufacturing techniques based on the filament extrusion method (MEX). Furthermore, the development of this particular method is driven by the widespread availability of this printing technique, particularly for non-professional users, hobbyists, and DIY enthusiasts. At the current stage of development of AM techniques, the ability to produce geometric models is no longer a major problem, even for very cheap desktop devices that offer excellent quality and repeatability of production [12,13]. Currently, many companies operating in the AM market are focused on the development of new types of multi-color or multi-material products, which makes the desktop 3D printing more useful, but also makes these machines more complex. The second trend applies to the production of technical products, where, due to low strength and thermal resistance, basic materials such as PLA or PETG do not allow for obtaining the desired properties [14,15,16,17,18,19,20,21]. Therefore, it is currently quite a big challenge to develop filament varieties with better functional properties. Current practice indicates the possibility of using two strategies to improve the mechanical performance or thermomechanical properties of the filament materials. In the first one, it is possible to use high-crystalline polymers; in this case, the most popular method in the MEX technique is the use of polyamide-based materials (PA12 or PA6) [22,23,24,25,26]. The second popular concept is the use of amorphous polymers characterized by a high glass transition temperature, where the commonly used type of polymer is polycarbonate (PC) [27,28,29,30,31,32]. In both cases, a typical limitation is the need to use a high bed table temperature and a heated working chamber, which is not standard in low-budget devices. The optimum solution is the preparation of polymer blends, which has been a common solution since the beginning of the plastic industry [33,34,35,36]. 

The material extrusion 3D-printing (MEX), also called the fused deposition modeling process (FDM), cannot be compared with the other thermoplastics shaping methods, since, for the mass scale processing, the geometry of the manufactured part is reproduced by the mold or die shape, where, due to high pressure and/or rapid cooling rate, the impact of material shrinkage is effectively minimized [37,38,39,40,41,42]. For the MEX processing method, the shape of the 3D model is reproduced layer by layer, where the melted materials are cooled down to the solid state temperature, without being kept in a mold where constant thermal conditions would prevail from deformation. It is therefore advisable that the properties of the materials used in MEX printing ensure minimal density change in phase transitions during cooling. Amorphous materials are a desirable solution here, for example, the widespread use of PS and ABS [43,44,45,46,47,48,49,50,51], while in practice, amorphous or slowly crystallizing thermoplastic polyesters, like PETG, PLA, and PET, are used much more often. In current practice, none of the popular varieties of polymer blends have found widespread use in 3D printing. Even popular materials like PC/ABS-blend systems are rarely used in additive manufacturing, where the main limitation is related to the high glass transition temperature for these blends, which standard 3D-printing machines are not suited for processing. The two-step strategy presented in this paper, which involves the use of a combination of two varieties of thermoplastic polyesters, one fully amorphous and the other with a semi-crystalline component, has already been used in research [52,53,54,55]. The annealing procedure is, of course, an alternative way to improve the thermomechanical properties of such a system [19,56,57].

The technological tests described in the present article aim to further develop this concept by using composite additives. The use of the talc/CF fillers is intended to achieve the typical reinforcing effect resulting from the introduction of rigid filler particles, but also to test the effectiveness of changes in the crystal structure formed with the help of fillers, which in turn may have a key impact on thermomechanical properties. In the case of previous studies, the use of talc proved to be a very effective method for achieving high efficiency of the crystalline phase nucleation process for materials such as PLA or PET [58,59,60,61,62], which also proved effective during the annealing procedure above the glass transition temperature [63,64,65]. For most of the conducted studies, the research works were carried out using small-sized specimens, mostly foils, which, when compared to industrial processes, cannot be considered a reflection of the phenomena occurring during injection molding or 3D printing. However, selected studies do elucidate certain phenomena that can be exploited in industrial practice to benefit the properties of materials. Mineral particles, as a commonly used additive, turn out to be a very effective material that can successfully support the efficiency of synthetic nucleating agents [66,67]. In recent years, a large part of the research has focused on the phenomena related to the thermal treatment of PLA with the addition of mineral fillers of waste origin, such as basalt, vermiculite or perlite. That favorable behavior mostly applies to systems where the properties of the final product are influenced by the morphology of the crystalline phase, which is not necessarily possible for the usually amorphous structure of additively produced products, in which case the most common solution turns out to be the use of fibers. In the case of current scientific research, there are numerous examples of the use of natural, basalt, aramid, or nanofibers [68,69,70,71,72,73,74]. In many of these cases, it is crucial to use fibers of appropriate length, preferably continuous, which is not possible with standard filament printing techniques and requires machines with advanced design. Taking into account the industrial solutions in the form of commercially available filaments, the most common use is short carbon fiber [75,76,77,78,79,80]. It is worth pointing out that the cited works clearly indicate the key role of the length of the fibrous structures on the expected properties of the products, where, in the case of the standard technique of compounding cut fibers and polymer granulate, obtaining properties similar to those of composite laminates is physically not possible. In the case of the discussed studies, the selection of fillers was aimed at assessing the effectiveness of changes in the structural properties of two commonly used composite additives. In the case of talc, the primary research objective was to assess the influence of this material on the crystalline structure of the rPET phase and the potential effects of these changes on thermomechanical characteristics. For modifications using CF, a more general assessment of the impact of this composite additive on the properties of the prepared mixtures is indicated.

The purpose of this research is to develop polymer composites designed for 3D printing. The base material in the modification process was recycled polyethylene terephthalate (PET), specifically rPET obtained from bottle recycling. Two types of copolymers, PETG and PCTG, were also included as components in the mixture. Both materials are amorphous thermoplastic polyesters, commonly used in 3D printing, making their addition significant for improving rPET processability. A fixed 50/50% ratio was maintained for the main components of the materials being developed, so the base for all materials consists of rPET/PETG (50:50) and rPET/PCTG (50:50) blends. Talc (T) and ground carbon fiber (CF) were used as polymer fillers.

Preliminary tests revealed very unfavorable mechanical characteristics for the processed systems, primarily high brittleness. These factors prevented the production of a continuous spool of filament, and injection molding attempts were also unsuccessful. In the second phase of development, the polymer matrix was additionally enriched with an elastomeric component (POE-g-GMA). To improve phase compatibilization, a reactive extrusion technique was used, incorporating a SAN-g-GMA chain extender. Modification of the materials prepared in the second phase was successful, allowing samples to be prepared using both injection molding and 3D printing. Research conducted to evaluate the material properties included basic mechanical measurements, thermal analysis using DSC and TGA, and thermomechanical testing using DMTA and HDT. Microscopic observations and rheological test results supplement these measurements. 

## 2. Experimental Section

### 2.1. Materials

The prepared materials consist of recycled poly(ethylene terephthalate) resin rPET, which was supplied by the company EUROCAST (Strzebielino, Poland). This particular type of rPET was made from the recycling procedure of PET bottles, and can be considered a post-consumer material type. The polyethylene terephthalate-glycol PETG copolymer was prepared from the unmodified filament, the trade name PETG Pure. Before the blending procedure, the filament was pelletized, which also applies to the polycyclohexylenedimethyl terephthalate-glycol PCTG resin, under the trade name PCTG Natural, in the preparation stage. Both types of amorphous polyesters were supplied by the company Fiberlogy (Brzezie, Poland).

**Figure 1:**
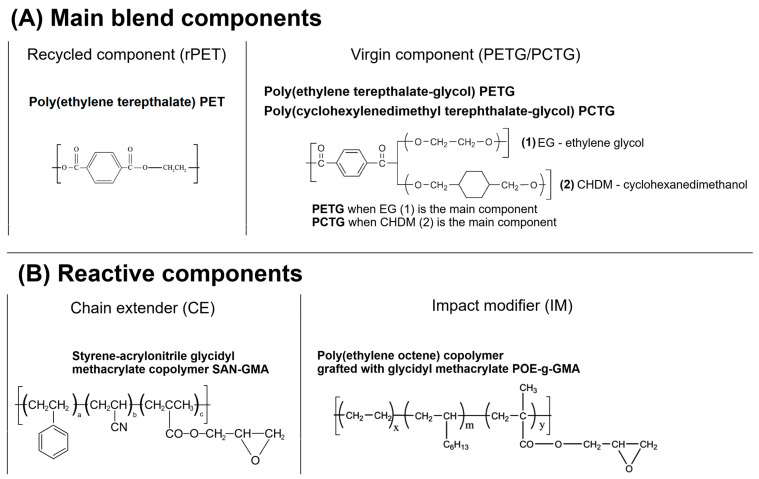
The chemical structure of the (**A**) main polymer components, and (**B**) reactive components used during the study.

The blends/composites were additionally modified using an elastomeric impact modifier (IM); the used compound was polyolefin-based copolymer functionalized with glycidyl methacrylate (POE-g-GMA), grafting level of 2–3%, MFR = 3–6 g/10 min (190 °C/2.16 kg), density of 0.89 g/cm^3^. This material was supplied in the form of transparent pellets under the trade name SOG-03 (Fine-blend, Shanghai, China). The reactive extrusion process (compatibilization) was performed with the presence of oligomeric chain extender, styrene-acrylonitrile glycidyl methacrylate (SAN-g-GMA), trade name SAG-08 (company Fine-blend, Shanghai, China). The chemical formulation of the used compound is presented in Figure 1.

**Figure 2:**
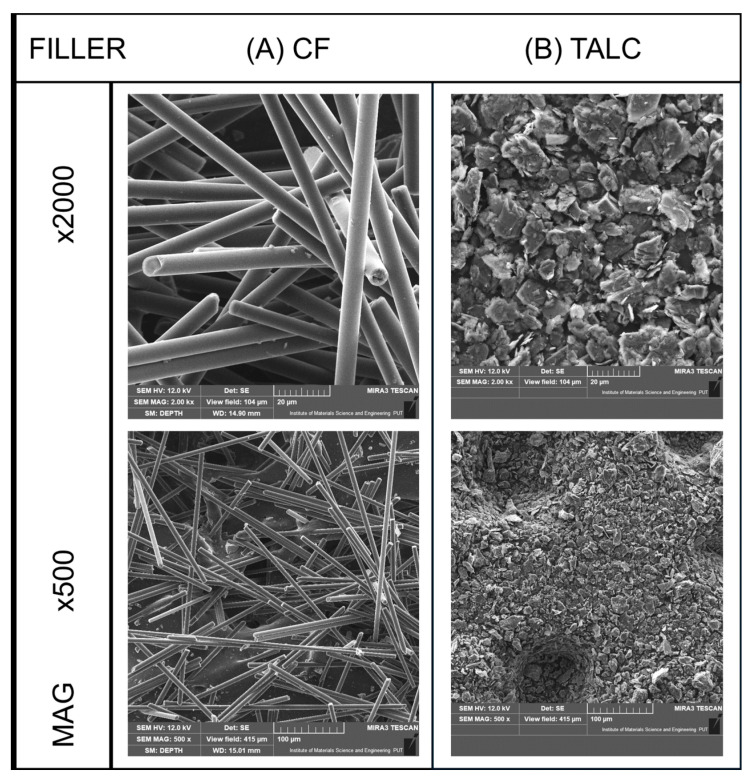
The scanning electron microscopy presentation of filler particles: (**A**) Carbon fiber; (**B**) talc.

The composite samples were prepared with the addition of talc filler (hydrated magnesium silicate), where the material was supplied in the form of white powder (company Biomus, Lublin, Poland). The average particle size for the talc filler was around 14 µm. The second type of filler consists of recycled carbon fiber (CF), Sigrafil C M150-3.0/200-UN (from SGL Carbon, Wiesbaden, Germany). The fibers were supplied in the milled form, where the average fiber length was around 150 µm, with a diameter of 7 µm. The SEM pictures revealing the appearance of the filler particles are presented in Figure 2.

The polymeric materials in granular form were dried before the process to minimize the effect of moisture on the material properties. The drying process was carried out in a chamber dryer at 80 °C for 12 h.

### 2.2. Sample Preparation

The sample preparation consists of a preliminary melt-blending procedure using a twin-screw extruder model EH16.2D (Zamak Mercator, Skawina, Poland). The extrusion process parameters were identical for all prepared materials. The maximum extrusion temperature at the die head was 255 °C; the temperature profile along the barrel ranged from 250 °C to 220 °C (feeding zone). The extruded material was cooled down using the air-cooled conveyor and pelletized. Before each stage of processing, the pellets were dried in a cabinet oven (12 h, 80 °C). The list of compounded samples is collected in Table 1, together with the material formulation. The prepared pellets were then used in the filament extrusion and injection molding processes. Since the preliminary set of materials was characterized by high brittleness, the filament extrusion and injection molding procedure was not possible; for this reason, the study was limited to DSC and rheological analysis, while the full set of testing procedures was performed on modified materials.

The filament extrusion was conducted using the single screw extruder, model Metalchem W25-30D (IMPiB, Torun, Poland), screw diameter of 25 mm, and L/d ratio of 30. The extrusion temperature measured at the die-head was 245 °C. The extruder die was equipped with a 2 mm diameter cylindrical nozzle. The screw speed during the filament extrusion ranged from 18 to 22 rpm, depending on the resin type. The extruded material was collected using a dry conveyor. The filament was cooled down with the air fans installed along the conveyor belt. To control the diameter of the filament rod (≈1.75 mm), the pulling system speed was changed from 5 to 8 m/min. Finally, the prepared filament was collected into separate spools using the dedicated winding machine. 

**Figure 3:**
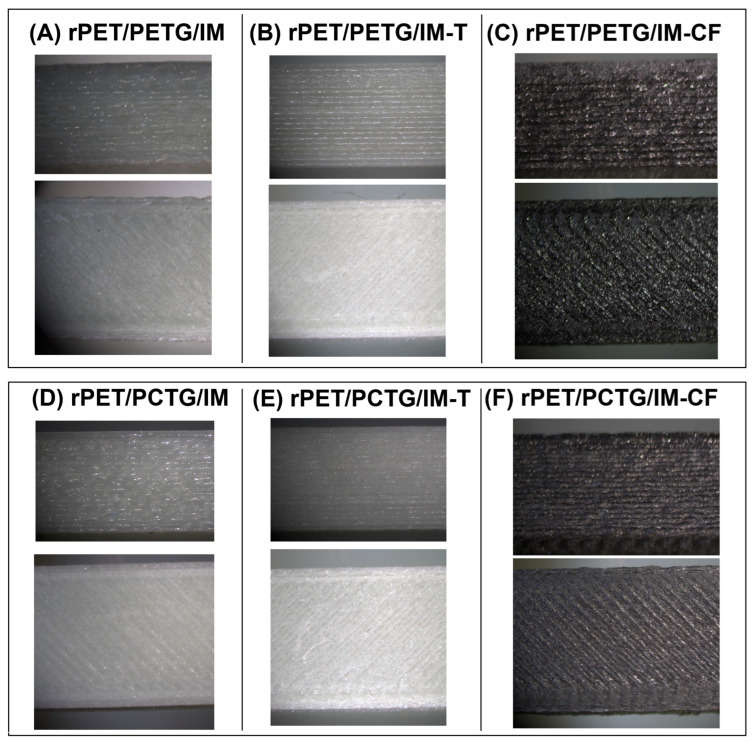
The enlarged view of the 3D-printed materials. Includes side and top views of the samples.

The 3D-printing process was conducted using a desktop-type Prusa MK3S machine (PrusaResearch, Prague, Czech Republic). The machine was equipped with a 0.4 mm diameter brass nozzle mounted on a direct drive extruder. The bed table was heated to 90 °C, while the surface of the platform table was covered with an adhesive layer (Dimafix glue). The nozzle temperature was set to 280 °C. The printing speed was set to 20 mm/s for the outer shell layers, while the infill structure was prepared at 50 mm/s. All specimens were printed with a 0.2 mm layer thickness. The machine code (G-code) files were prepared using PrusaSlicer 2.2.1 software; the input STL files were prepared using Fusion 360 software (Autodesk, San Francisco, CA, USA). The general appearance of the sample structure, taken from top and side views, is presented in Figure 3.

Injection molding was used as the reference technique for the MEX printing procedure. For this purpose, we used the Engel Victory 50 molding machine (Schwertberg, Austria). The injection machine was equipped with a 25 mm diameter screw and a 500 kN hydraulic press. For the purpose of the study, we used a water-cooled mold, in which the mold cavity allowed simultaneous shaping of dumbbell samples and rectangular bars. The molding procedure was conducted at the maximum temperature of 260 °C, measured at the nozzle. The injection pressure was set to 800 bar, with a holding pressure of 400 bar. Cooling time was 40 s. Mold temperature was set to 30 °C.

**Figure 4:**
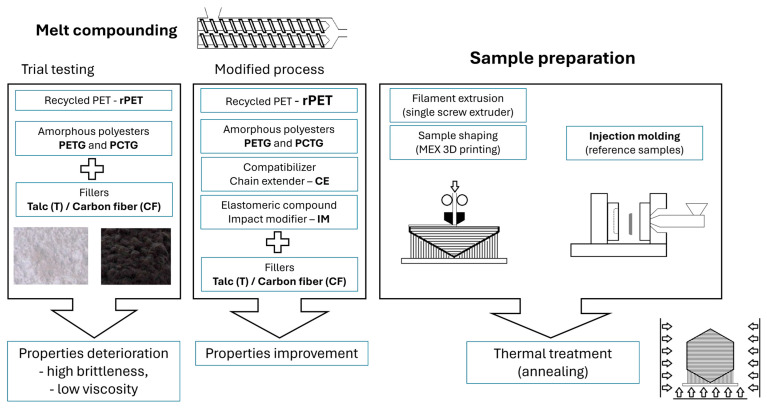
The sample preparation procedure, including the melt compounding process and the sample shaping/preparation concept.

The annealing procedure was conducted for both injection-molded and 3D-printed specimens, where the selected specimens were placed inside the heated chamber of the oven dryer. The temperature of the annealing procedure was set to 150 °C, the samples were kept at the maximum temperature for 3 h, and then slowly cooled in the oven for about 2 h. The scheme presents the research procedure in Figure 4.

### 2.3. Characterization

The microscopic observations were performed using the scanning electron microscopy method. The cross-section samples were prepared from the fractured impact test specimens, where the surface was coated with the conductive layer of carbon using a vacuum sputter (model Jeol JEE 4B, Tokyo, Japan). For the filler samples, the particles were placed on the glued surface of the microscope table before coating. For all observations, we used the Tescan MIRA3 SEM microscope (Brno, Czech Republic).

The thermal properties of the prepared samples were investigated using the differential scanning calorimetry (DSC) method. The measurements were carried out using a heat-flux-type apparatus, model Netzsch DSC 214 Nevio (Selb, Germany). The samples used during the study were placed in an aluminum crucible. Tests were performed using a standard 3-stage program (heating/cooling/heating), and the heating/cooling rate was set to 10 K/min. The temperature of the measurement ranges from 20 °C to 300 °C. The crystallinity level was calculated according to the following Formula (1):

(1) $$\%\;Crystallinity\;=\;X_{c}\;=\;100\;\times \;\frac{\Delta H_{m}-\;\Delta H_{cc}}{\Delta H_{100}\;\left(1-\varphi \right)}$$

where ∆*H_m_* is the measured melting enthalpy, ∆*H_cc_* is the measured enthalpy of cold crystallization, and Δ*H*_100_ is the theoretical melting enthalpy, *φ* refers to the filler/additive content. The melting enthalpy for the 100% crystalline PET phase was taken from the previous literature [81,82,83].

Mechanical performance was investigated using the static tensile method, where tests were conducted according to the ISO 527 method [84]. The Charpy impact resistance measurements were performed using notched samples, according to ISO 179 standard [85]. The tensile measurements were conducted using the Zwick/Roell Z010 machine (Zwick/Roell GmbH, Ulm, Germany). The type 1A specimens were tested at 10 mm/min. The impact tests were performed using the HIT 25P hammer (Zwick/Roell, Ulm, Germany) machine, which was equipped with the Charpy type pendulum (5 J).

Dynamic mechanical thermal analysis (DMTA) was conducted using DMA 242 E Artemis apparatus (Netzsch GmbH, Selb, Germany). The machine was equipped with a dual cantilever clamping system, where tests were conducted using a 40 µm deformation amplitude. The deformation frequency was set to 1 Hz, while the heating rate was 2 K/min. The measurement temperature ranges from −50 °C to 200 °C. Thermomechanical studies were supplemented with heat deflection temperature (HDT) and Vicat softening temperature (VST) measurements. For this purpose, we use the RV300 Vicat/HDT apparatus (Testlab, Warsaw, Poland). The HDT measurements were performed according to the ISO 75 standard [86] using two testing loads, 0.455 MPa and 1.8 MPa. The VST tests were conducted in accordance with the ISO 306 standard [87], where 10 and 50 N loads were used. For both HDT and VST measurement, the heating rate was set to 2 K/min.

Rheological analysis was conducted using a rotational rheometer model MCR 102 (Anton Paar GmbH, Graz, Austria). The apparatus was equipped with a parallel plate system with a 25 mm diameter. The measurement gap was set to 1 mm. The main viscosity measurements were conducted using the oscillatory frequency-sweep method, while the linear viscoelastic region was determined using amplitude-sweep tests. For the main testing, the temperature was set to 270 °C. The measurements were conducted under a protective nitrogen atmosphere.

The study employed a handheld, metrological laser scanner, the HandySCAN BLACK Elite, manufactured by Creaform (Lévis, QC, Canada). The device operates based on laser triangulation technology with dynamic positioning relative to reference markers, enabling precise geometry acquisition without the need for an external tracking system. The scanner features a single-point accuracy of ±0.025 mm and a volumetric accuracy of 0.020 mm + 0.040 mm/m (VDI/VDE 2634 Part 3). The measurement resolution of the sensor is up to 0.025 mm. Prior to the measurements, the system was calibrated according to the manufacturer’s procedure. Reference markers were distributed over the surface of the tested objects to enable real-time tracking of the scanner position. During data acquisition, the aperture was set to 1.0, allowing optimal adjustment of the optical exposure to the reflective properties of the scanned surface. Geometry reconstruction was performed using a mesh resolution parameter set to 0.4 mm. The resulting measurement mesh was subjected to comparative analysis by aligning it with the reference CAD model. The alignment process was carried out using a least-squares deviation minimization algorithm. Based on this procedure, surface deviation maps were generated, and the maximum, minimum, and mean geometric differences between the measured model and the reference model were determined.

## 3. Results and Discussion

### 3.1. Macro/Microstructure Observations—SEM Microscopy

**Figure 5:**
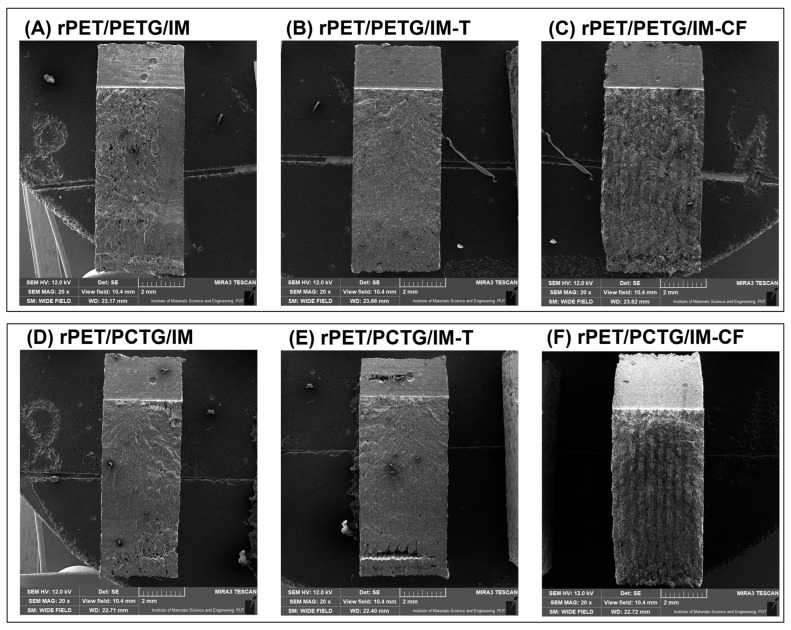
The appearance of the fractured impact test specimens for: (**A**,**D**) unmodified blends; (**B**,**E**) talc(T)-loaded materials; (**C**,**F**) carbon fiber (CF)-loaded materials.

**Figure 6:**
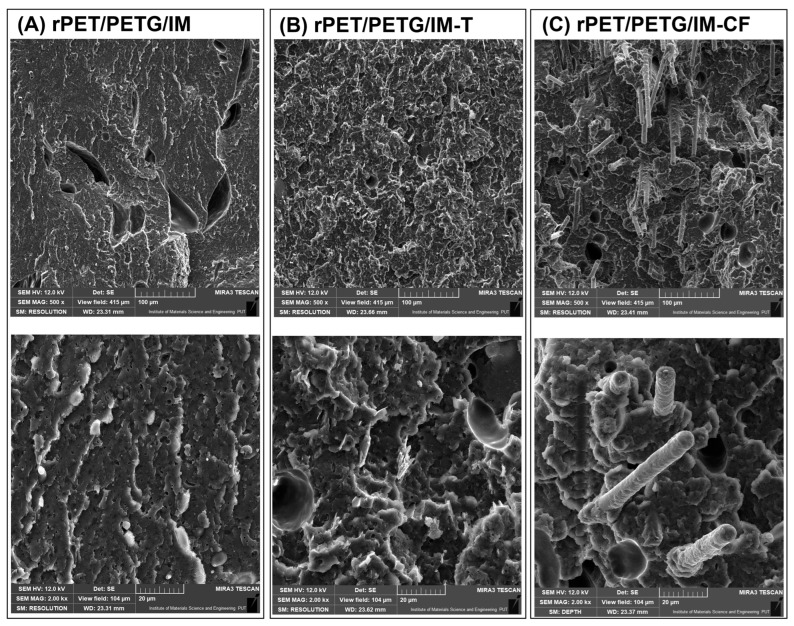
The SEM analysis for the PETG-based materials: (**A**) rPET/PETG/IM blend; (**B**) rPET/PETG/IM-T composite; (**C**) rPET/PETG/IM-CF composite.

**Figure 7:**
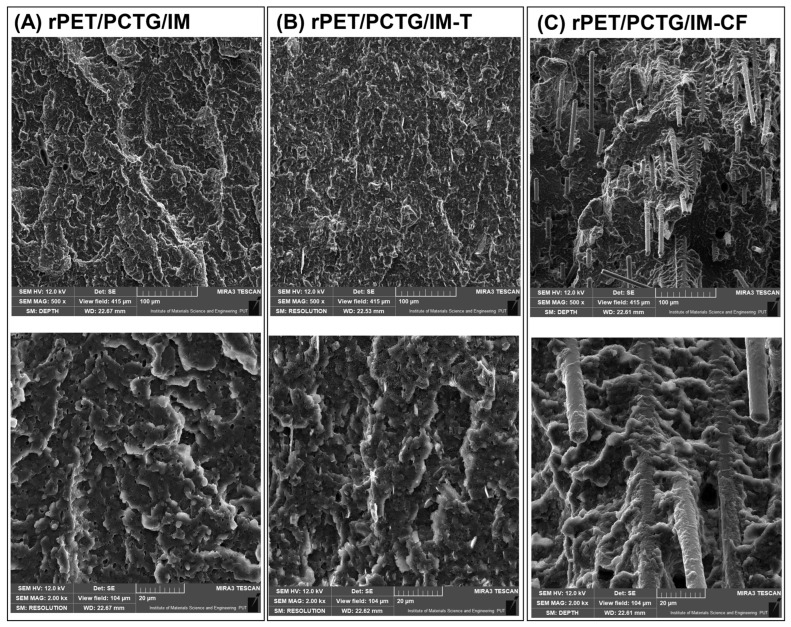
The SEM analysis for the PCTG-based materials: (**A**) rPET/PCTG/IM blend; (**B**) rPET/PCTG/IM-T composite; (**C**) rPET/PCTG/IM-CF composite.

The scanning electron microscopy observations were conducted for the fractured surface of the MEX-printed specimens. Macroscopic images of the inspected samples are presented in Figure 5, while a more detailed microstructural analysis is presented separately for the PETG-based (Figure 6) and PCTG-based materials (Figure 7). The sample structure at the macroscopic scale reveals a semi-solid structure in the prepared specimens, which is a result of the used infill density of 100%. The layered structure of the specimens prepared from the unloaded blends is difficult to distinguish. The reason for that might be related to the machine code settings, where the extrusion flow rate was set to 100% for all samples, while in most cases this parameter reached 90–95%. For the composite specimens, the individual layer structure is more visible, which might be related to the less compact structure of the Talc/CF modified materials. Additionally, the fibrous structure of the CF-modified materials can be easily recognized even at low magnification (20×).

The microscopic scale view of the unloaded rPET/PETG/IM blend reveals a very homogeneous dispersion of the elastomeric domains. Interestingly, the scanning microscopy method is not a perfect tool for blend miscibility evaluation, since, except for the presence of the POE-g-GMA, no other inclusions are detected. The same conclusion applies to the rPET/PCTG/IM blends, where the same homogenous sea-island structure is observed [88,89,90]. The presence of talc particles did not lead to large structure differences. Since the filler content was calculated using the weight ratio (10% wt.), the real volume content of the particles is around 4.9%, which is related to the talc density of 2.58–2.83 g/cm^3^ [91,92]. The presence of talc particles is therefore revealed in a relatively small percentage; however, considering the homogeneity of the prepared polymer system, the particles are well dispersed, with no agglomeration tendencies. Additionally, the structure analysis revealed that the talc particle size was visibly reduced compared to the unprocessed filler. This observation can be related to the lamellar structure of the talc filler, where the high shear forces during flow processes lead to a reduction in the dimensions of the talc plate diameter. Taking into account the low filler content, changes in the morphology of the particles themselves do not have a significant impact on the structure’s appearance; however, considering the possible use of nanosized mineral particles, this aspect can be more important for future research. 

The presence of the CF filler is much more evident, since even the macro view revealed the presence of fibrous reinforcement, unlike the talc particles, where the presence of filler particles was relatively small. For milled carbon fibers, the observed structures were significantly influenced by the filler presence, which was only partly caused by the lower density of the CF filler (1.8 g/cm^3^) and related volume content of around 7.4%. For both PETG and PCTG-based blends, the dispersion of CF is homogenous, which means that most of the fibrous particles are well embedded in the matrix. Additionally, the composite interface analysis revealed very good adhesion between both types of matrix blends and the CF surface. None of the prepared scans revealed the presence of an interface gap at the phase boundary. Interestingly, even the surface of torn fibers is covered with a layer of matrix material. It is worth noting that the surface of raw carbon fiber is difficult to modify; modification is typically achieved using various surface oxidation techniques [93,94,95]. For the milled fibers that were used during the discussed study, the important factor that might explain the observed behavior is the use of pyrolysis techniques during the recycling procedure [96,97,98]. Surface phenomena related to wear during grinding and processing of fibers lead to damage that would lead to a weakening of the strength of the products in the original fiber processing processes. In the case of milled fibers, which constitute waste during the recycling of CF composites, due to the significant reduction in the fiber length, their structural uniformity and strength are no longer important, which makes the material perfect for techniques such as 3D printing; they demonstrate very high reinforcement efficiency. 

The previous research on polyester mixtures allows for the possibility of a two-phase structure of these materials [20,99,100]; however, the available SEM imaging does not allow for the identification of such areas, which confirms that the tested PET/PETG and PET/PCTG systems are miscible, at least partially. Unfortunately, due to the overlapping glass transition ranges of the components in the prepared mixtures, DSC or DMA analysis results do not allow for the assessment of the compatibilization/miscibility of the system, which can be easily performed for different types of polymer blends, such as PC/ABS, and PC/PBT [101,102,103,104]. In the case of analyses conducted where the main focus of the research was on further modification of these types of materials, it seems pointless to further analyze the structure of the mixture itself, either using AFM or TEM microscopy. However, the issue itself, due to its fundamental impact on the properties of these types of materials, is still worthy of separate analysis.

### 3.2. Thermal Properties/Crystallinity/Phase Transitions—DSC Analysis

**Figure 8:**
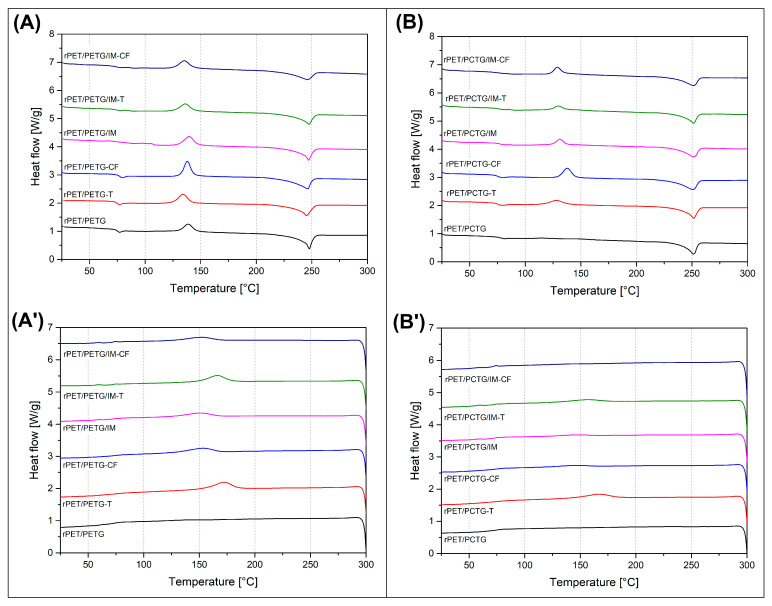
The DSC thermograms reflecting the thermal properties for (**A**,**A’**) PETG-modified materials, and (**B**,**B’**) PCTG-based samples.

**Figure 9:**
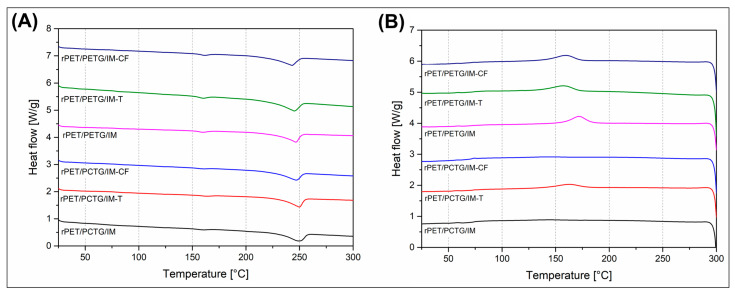
The DSC thermograms for annealed materials: (**A**) 1st heating scans; (**B**) cooling scans.

The results of the DSC analysis are presented in the form of thermograms (Figure 7 and Figure 8), while some of the basic data are also collected in Table 2. Since the DSC method does not require a standardized sample geometry, measurements were performed on all melt-blended materials, including those characterized by high brittleness. For this reason, the samples prepared without the impact modifier (IM) addition were cut from the compounded pellets, while the rest of the materials with the IM addition were collected from the MEX-printed materials (see Figure 8). The additional analysis was conducted on the annealed materials, with all specimens cut from the MEX-printed materials (see Figure 9). The plots collecting the thermograms for the reference materials are presented in Figure S1 (Supplementary Information Section).

The DSC thermograms of the rPET/PETG blends exhibited typical behavior, with the thermograms primarily reflecting the thermal transitions of the PET phase. Since the only noticeable signal change for the PETG component was the glass transition T_g_ (see Figure S1), the transition signals are overlapping. For the rest of the thermogram range, the observed signal changes are referred to the PET phase. For all of the investigated samples, the initial cold crystallization peak position ranges from 135 °C to 140 °C, which is in line with most of the previously reported results for PET-based blends [105,106]. The same conclusion is regarding the melting peak area, where for most of the samples, the results are close to 250 °C, with small deviations of around 5 °C. The fluctuations can be associated with some slight differences in lamellar structure thickness; however, due to the rather random nature of these small differences, it is not possible to correlate these changes with blend composition. More interesting analysis can be made when taking into account the peak area comparison and the resulting crystallinity level calculations. The reference value for the unmodified rPET resin was close to 13%, results of which is strongly in line with the reported results for recycled PET [107,108]. The addition of PETG/PCTG amorphous component strongly improves the PETG phase crystallinity, while the calculated values reached 26.3% and 30.5%, respectively, for rPET/PETG and rPET/PCTG blends. The discussed materials were prepared using the standard melt-blending procedure without any reactive additives that might improve the molecular weight or macromolecular entanglement. This means that the enhanced crystalline phase formation can be associated with the reduction in the molecular weight for the PET phase and/or the formation of the immiscible double phase blend structure. For the prepared materials, the previous studies revealed that both PETG and PCTG resins are miscible with the PET phase [53], which was confirmed by a homogenous structure and lack of visible phase separation. In principle, such information does not exclude the formation of areas with a higher density of individual phases, which can lead to the formation of a partly heterogeneous structure and induce the nucleation phenomena of the PET crystal structure. Interestingly, the formation of the crystalline structure was slightly reduced for the materials with filler additives. The addition of talk (T) and carbon fiber (CF) reduced the crystallinity to 20 (±3) %, with slightly higher values for talk filler compounds. Usually, in semicrystalline polymers, the addition of solid fillers improves crystallinity [109,110,111], whereas in the investigated CF/talc-loaded materials, crystallinity decreased. That kind of behavior proves that for the slowly crystallizing polymers, the formation of the ordered lamellar structures is strongly dependent on the blend composition, the used additives, and the cooling conditions. The processing conditions for all samples prepared during the preliminary stage of the research were similar, since the DSC specimens were cut from the compounded pellets. It means that the cooling rate was relatively high compared to compression molding or injection molding at high mold temperatures. Considering all the mentioned factors, the possible reason for the observed differences is the change in thermal conductivity caused by the filler addition, a phenomenon which was already observed for other types of materials [112]. Interestingly, for impact-modified materials, the introduction of another polymeric phase did not lead to a visible difference in DSC signals and crystallinity level. In this particular case, the used elastomeric compound was the functionalized POE-g-GMA copolymer. For the rPET/PETG/IM samples, the crystallinity level ranged from 16% to 20%, while for the rPET/PCTG, it was 21–27%. Interestingly, for none of the prepared materials, the addition of filler particles (CF/talc) or elastomeric phase did not increase the crystallinity level significantly, despite the additional changes in the thermal history. Considering the possible crystallinity for the PET phase, which can reach around 60% after annealing, it is worth noting that before heat treatment, the macromolecular structures of the PET phase were mostly amorphous. In addition, it is worth noting that the conducted DSC study did not confirm any significant influence of the filler or impact modifier addition; the reason for that might be associated with the complex nature of the prepared blends, where the crystallinity calculations were performed for the semi-crystalline rPET phase. The maximum weight content of the PET phase was 50%, while the materials with the addition of impact modifier and filler particles were limited to 36%, which additionally makes it difficult to analyze the obtained results. Additionally, samples prepared at the preliminary stage (without impact modifier-IM) were cut from the extruded pellets, while at the main stage of the research (with IM compound), specimens were cut for the already printed parts, which is another factor that influences the thermal history of the formed macromolecular structure.

The analysis of the cooling stage thermograms revealed the thermal behavior of the PET phase during the formation of the crystalline phase. Since the structure was remelted at 300 °C during the 1st heating stage, the cooling scans reflect the changes in crystallization kinetics more properly. Interestingly, for the unmodified blends for both PETG and PCTG-based materials, the cooling scans revealed lack of exothermal peak, which is in contrast to the unmodified rPET sample, where a strong peak was recorded at 194 °C. That fact confirms the presence of strong intermolecular entanglement or miscibility for both rPET/PETG and rPET/PCTG blends, where the formation of the PET lamellar structures is hindered by the presence of the secondary phase polymer chains. The amorphous nature of the blend structure is observed only for the unmodified samples, as each of the remaining DSC graphs confirms the formation of lamellar structures by a noticeable exothermic peak. It is interesting, however, that for rPET/PETG materials the peak size is visibly larger, which suggests a much more intense kinetics of the crystallization process, while for some of the rPET/PCTG samples the graph is almost flat with a very low DSC signal value. Analysis of the graphs also indicates an increase in the crystallization temperature for the sample with talc added, where the range of values is from 158 to 172 °C, while for the remaining materials, including the sample with CF added, it is approximately 147–154 °C, which confirms the high nucleation efficiency for talc particles [113,114,115]. The surface of the carbon fiber fillers is usually less effective, which is confirmed for many types of semicrystalline polyesters [112,116,117].

The DSC analysis is supplemented with the measurements conducted for the annealing-treated specimens (see Figure 9). Since the 3D-printing procedure was possible only for impact-modified materials (IM), the DSC analysis is limited to the MEX-printed/annealed specimens, which limits the number of samples. The resulting 1st heating plots reveal the presence of a highly crystalline PET phase structure, which is confirmed by the lack of cold crystallization signal for the annealed materials. The crystallinity level calculations revealed that the reference value of 55% for the rPET/PETG/IM sample was strongly improved after the introduction of filler, up to around 70% and 63%, respectively, for talc (T) and CF filler. The same crystallinity level factor for rPET/PCTG/IM samples was more stable, since the reference value of 50% for the unloaded sample was slightly increased to 56% (talc) and 52% (CF). The recorded results are strongly in line with the previously reported values for similar types of materials, which confirmed the efficiency of the conducted heat treatment [118,119]. Interestingly, for all of the investigated samples, the analysis revealed the presence of a small endothermic peak at around 160 °C. This kind of phenomenon confirms the presence of a small amount of PET phase crystalline structure. Due to rapid cooling, the size and level of order of this type of formation are low, hence the melting occurs at low temperatures close to the annealing temperature.

The cooling scans for the annealed samples are revealing some similar trends already recorded for the untreated materials. In PETG-based samples, the crystallization peaks are more distinct; however, the peak positions are relatively random, suggesting a lack of highly efficient nucleation of filler particles. It is worth noting that the described results mostly focus on the analysis of the thermal behavior for the PET phase, which is only one of the blend components, where the second, no less important, is the amorphous copolymer (PETG/PCTG). Considering that the obtained blend structures are miscible, the resulting features of the crystalline PET phase can be strongly influenced by the presence of strongly entangled amorphous regions, gradient structures, and content changes between rigid amorphous and mobile amorphous phase structures (RAF/MAF). For this reason, the incorporation of filler particles, like platelet talc (T) and fibrous carbon fibers (CF), is another factor that changed the already complex polymer system. This finally makes the interpretation of DSC results less precise. However, summarizing the conducted measurements, it can be stated that the annealing procedure strongly improves the PET phase crystallinity, which was the main reason for the thermal treatment process. 

### 3.3. Mechanical Performance—Static Tensile Tests/Charpy Impact Measurements 

**Figure 10:**
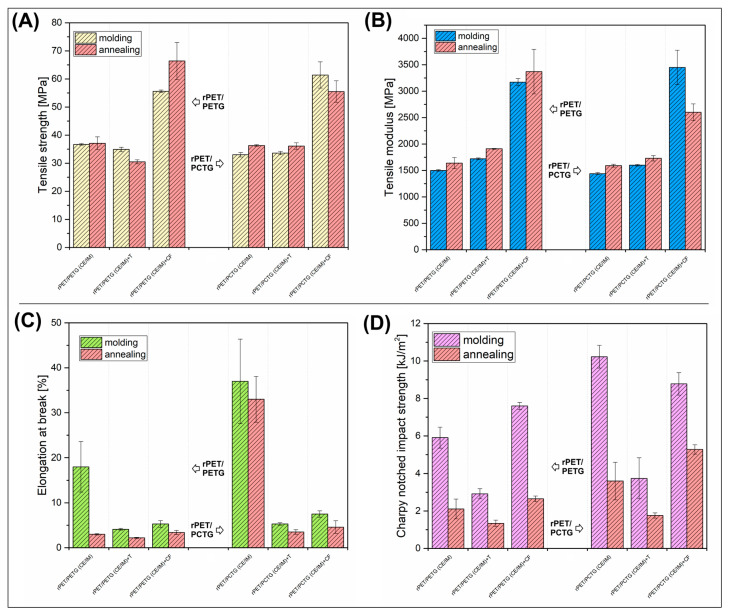
The results of the mechanical properties evaluation for injection-molded specimens: (**A**) tensile strength; (**B**) tensile modulus; (**C**) elongation at break; (**D**) impact strength.

**Figure 11:**
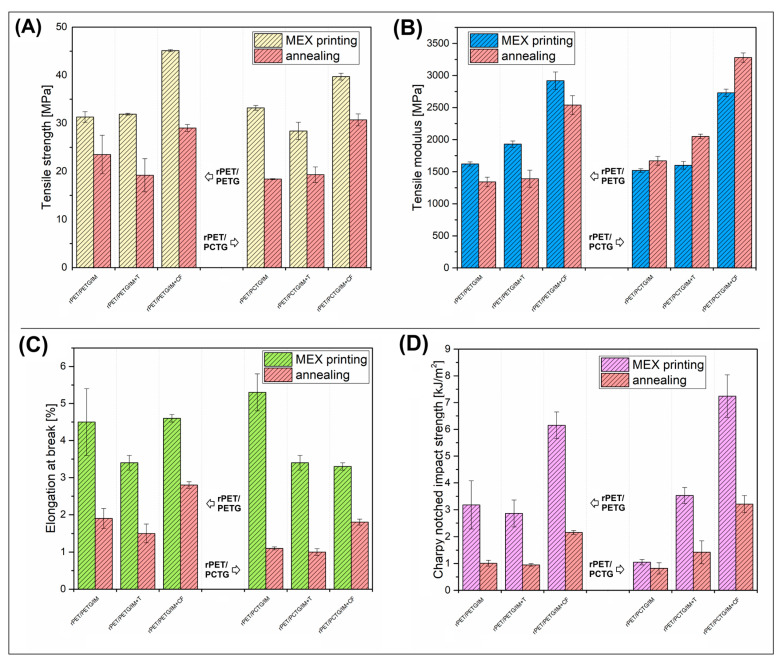
The results of the mechanical properties evaluation for MEX-printed specimens: (**A**) tensile strength; (**B**) tensile modulus; (**C**) elongation at break; (**D**) impact strength.

The mechanical properties of the prepared materials were evaluated using static tensile measurements and Charpy impact tests. The results are divided into two groups: the results for injection-molded samples are shown in Figure 10, while for MEX-printed samples, the data are shown in Figure 11. Additionally, all recorded values are presented in Table S1 (Supplementary Information Section). As usual, for the direct comparison, the mechanical properties of the injection-molded material are visibly more favorable compared to the MEX-printed counterparts, which is why the scalebars for both graphs are different, and the first and main assumption regarding the presented analyses confirms significantly lower values of mechanical characteristics for 3D-printed samples. However, most of the changes caused by the addition of fillers are similar in both types of materials, which suggests that the addition of talc (T) and CF has a significant effect on structural changes regardless of the manufacturing method. 

The tensile strength changes for molded specimens revealed a typical behavior of low filler-loaded composites (see Figure 10A). Since the content of the T/CF reached only 10% wt., the reinforcing efficiency was very small. Especially for the talc platelets loaded materials, the tensile strength was not changed, which was recorded for both the rPET/PETG/IM and rPET/PCTG/IM samples. In contrast, the addition of the CF filler visibly improved the strength factor, confirming that the fibrous particles are much more efficient as a reinforcement, even for materials with strongly reduced fiber length, such as the recycled CF used. Similar behavior was reported for MEX-printed materials (see Figure 11A), where again the CF filler was evidently more efficient as the structural reinforcement. In practice, the results confirm the validity of the common strategy adopted by filament manufacturers: instead of preparing highly loaded complex composites, using a small amount of CF-based filler yields the desired properties, primarily stiffness and strength. Interestingly, the comparison between the molded and annealed materials revealed that the annealing process was more strongly influencing the properties of the MEX-printed parts. For most of the injection-molded specimens, the tensile strength was only slightly changed, mostly improved, while the recorded values were often within the range of about 10%, which can be considered a negligible change. In contrast, for 3D-printed materials, the annealing visibly reduced the tensile strength, in the range of 30–40%. This behavior may confirm the occurrence of significant, larger changes in the failure mechanism for samples manufactured by 3D printing. In this case, for molded materials, an increase in crystallinity may lead to a slight increase in the stress at break for selected samples. However, for 3D-printed materials, these changes have a less significant impact, as the fracture mechanism is determined by phenomena occurring at the boundaries of the filament layers, where an increase in crystallinity proves less favorable.

The main trends observed for tensile strength are reflected during the analysis of the tensile modulus (see Figure 11B), for both PETG and PCTG-based blends, the stiffness of the reference blends of around 1500 MPa was slightly improved to 1600–1700 MPa after the addition of 10% wt. of talc (T) filler. The use of CF filler resulted in a greater increase, exceeding 3000 MPa. Interestingly, the results for the MEX-printed materials were reduced only for the CF-loaded samples, where the highest values reached 2700–2900 MPa. The stiffness of the reference blends and talc-loaded materials ranged from 1500 MPa to 1900 MPa, which was consistent with that of the molded samples. Interestingly, the comparative analysis for the annealed materials revealed some important differences between the investigated groups of materials. For the molded rPET/PETG/IM-based samples, the heat treatment led to a slight improvement in tensile modulus (≈5%), while when the same procedure was conducted for MEX-printed materials, it resulted in a visible decrease of about 15–20%. The same measurements of the tensile modulus for the rPET/PCTG/IM materials revealed that the modulus was improved for almost all of the investigated samples; the only exception was the molded rPET/PCTG/IM-CF sample. Such divergent results confirm that even seemingly simple relationships in the case of polymeric materials with different processing histories can constitute a very complex research problem, and the trends in property changes are not predictable. Sometimes, due to the multitude of factors, they take a random course of changes. Therefore, taking into account the observed differences in the strength/modulus changes, it is worth emphasizing several basic dependencies. The main factor that improved the tensile strength/modulus was the addition of CF filler. The influence of the annealing treatment was less favorable for MEX-printed materials, while the mechanical properties of the molded materials were changed only slightly. To explain some of these unexpected results in modulus decrease after annealing, we are considering the influence of the following factors: thermal relaxation, reduction in macromolecular orientation, and crazing at the layer bonding. None of these phenomena can be confirmed without additional deep investigation and a more directed sample preparation methodology. At the current stage of our project, we are performing a more focused study regarding the preparation of talc and CF-reinforced materials, where the annealing treatment will be conducted during the printing procedure. We think that this kind of study will be more helpful for the evaluation of the structure-properties correlations.

Since the exceeded brittleness of the trial test material was the main reason for working with elastomer-modified materials (IM), the elongation at break and impact resistance results are crucial, considering the possible applications. However, the results for elongation at break confirm that, even for the molded composite specimens, it was not possible to exceed 8% of the maximum strain, whereas for MEX-printed specimens, none of the talc/CF-reinforced samples exceeded 5%. A significant factor contributing to such low-test results is the relatively poor results for the unreinforced blend samples. The elongation at break for the rPET/PETG/IM material reached around 18%, while for the rPET/PCTG/IM blend it was 36%; these results partly reflect the maximum strain differences between the amorphous copolyester, where the results for pure PETG of 17% are much lower than for the PCTG, where the recorded values reached 160%. Due to the high fragility of the rPET sample, mechanical tests proved to be very difficult, which suggests that the used PET variety, which was a regranulate based on recyclates from PET bottles, was characterized by very low quality caused by an intensive process of degradation. Hence, for the blends produced in the first stage of the research, it was not possible to prepare the samples with appropriate strength. However, the key test samples were produced with a 20% IM addition, which generally allows for satisfactory results, at least for injected molded materials [21,53,120]. Unfortunately, for the prepared materials, both the waste origin of the rPET component and the addition of filler particles result in the obtaining of materials with a low elongation at break. The annealing procedure led to more unfavorable properties, as the maximum strain was strongly reduced in all prepared samples. An interesting exception is the unloaded rPET/PCTG/IM sample, where, despite using the same annealing procedure, the elongation still maintained the axis at a relatively high level of around 33%. Unfortunately, the result refers only to injection-molded materials, which, from the point of view of the conducted research, does not constitute a significant improvement. Therefore, taking into account the properties of the samples printed using the MEX technique, elongation measurements give unfavorable results. 

The impact resistance characteristic might be considered the most important one, since one of the main reasons for the use of elastomeric modifiers was the reduction in brittleness. The reference value for the rPET resin could not be measured, while for the used copolymers, the obtained results suggest more favorable properties for the pure PCTG resin. The recorded impact strength reached 12 kJ/m^2^ and 3 kJ/m^2^, respectively, for PCTG and PETG injection-molded samples. Such a difference for the main components of the mixture explains the differences obtained for complex systems, where the strength for the rPET/PETG/IM and rPET/PCTG/IM blend was 6 kJ/m^2^ and 10 kJ/m^2^, respectively. The addition of the filler particles reveals a large difference between the powder talc and the fibrous CF filler. For both types of talc-modified materials, the impact strength was reduced below 4 kJ/m^2^, while the results for CF-modified specimens were in line with the values recorded for unloaded blends. This type of behavior might be associated with the pull-out phenomenon observed for many types of carbon-fiber-based materials [121,122,123]. Interestingly, it is even more pronounced for the MEX-printed materials, where the results obtained for CF-loaded materials were in the range of 6–8 kJ/m^2^, while for the rest of the materials, the impact strength was below 4 kJ/m^2^, which suggests a lack of favorable properties again. The analysis of the impact resistance results after the annealing process revealed another reduction in material strength. The recorded values for CF-reinforced materials are close to 4 kJ/m^2^, which is very similar to that of brittle materials such as polystyrene or poly(lactic acid) [124,125,126,127]. Hence, a moderate conclusion is that all positive effects of the annealing process are simultaneously associated with a significant loss of elongation and impact strength. 

### 3.4. Thermomechanical Properties—DMTA/Heat Resistance Tests

**Figure 12:**
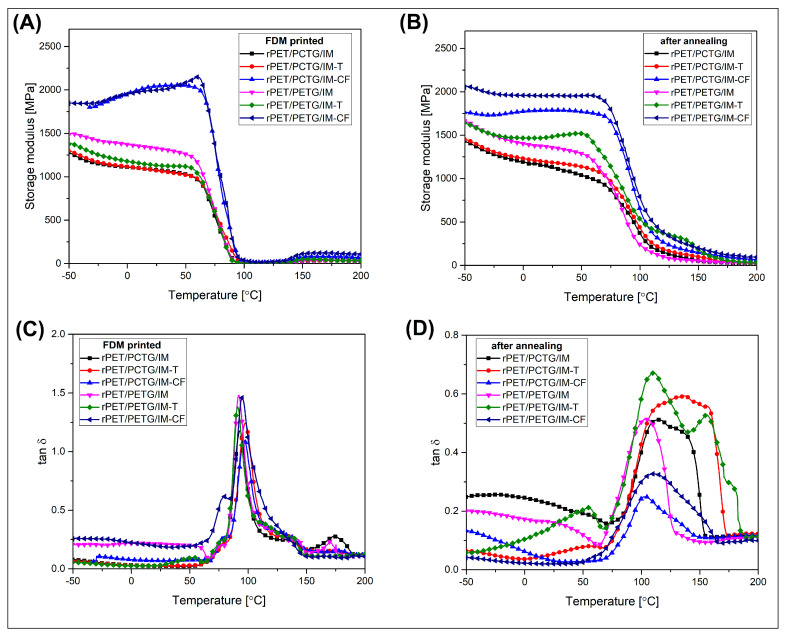
The viscoelastic properties obtained during the DMTA measurements, the storage modulus, and tan δ plots for: (**A**,**C**) MEX-printed samples, and (**B**,**D**) annealed materials.

**Figure 13:**
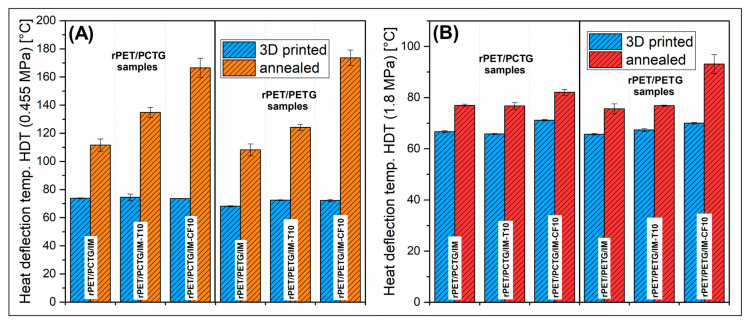
The results of the heat deflection temperature (HDT) measurements for MEX-printed and annealed materials: (**A**) load 0.455 MPa; (**B**) load 1.8 MPa.

**Figure 14:**
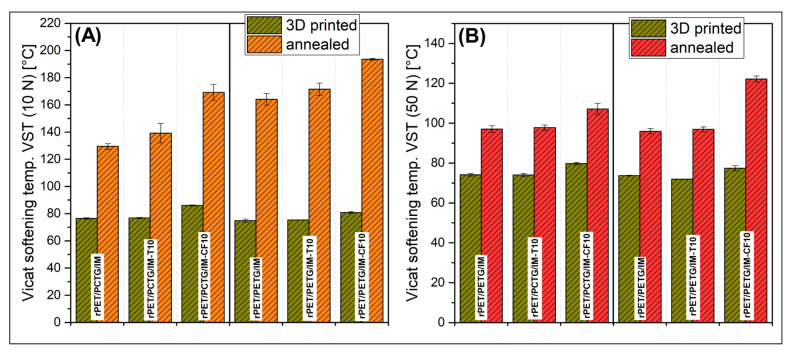
The results of the Vicat softening temperature (VST) measurements for MEX-printed and annealed materials: (**A**) load 10 N; (**B**) load 50 N.

The thermomechanical properties of the prepared materials were investigated using the dynamic thermal mechanical analysis method (DMTA) and the HDT/Vicat tests. The results for DMTA were presented in the form of storage modulus and tan δ plots (see Figure 12). The HDT test results are shown in Figure 13, while the Vicat softening temperature measurements are shown in Figure 14. Both DMTA measurements and HDT/Vicat tests were limited to MEX-printed specimens. 

The DMTA was conducted from −50 °C, the fact which could help with the evaluation of the possible changes in viscoelastic behavior at low temperature. It is worth noting that the glass transition of the used IM compound (POE-g-GMA) was around 58 °C, which means that the conducted analysis did not provide information regarding the phase transition of the elastomeric phase. However, from a practical point of view, the tested materials will not find application in cryogenic conditions, which is why the temperature range of the conducted study is sufficient. The storage modulus plots reveal that the stiffness measured at low temperature range was almost constant with a very small decreasing trend. The absolute values of the storage modulus for individual samples slightly differ, but the recorded changes for the reference materials and those with added talc were negligible and random in nature. However, the addition of CF filler strongly changes the plot characteristic. For both rPET/PETG/IM-CF and rPET/PCTG/IM-CF composites, the storage modulus was shifted to higher values, which confirms reinforcing efficiency. The storage modulus values are stable up to 60 °C, where for all of the investigated samples, the glass transition phenomenon leads to a visible drop in stiffness. For all of the prepared materials, the structure softening process leads to a very rapid loss of mechanical properties. When already at around 100 °C, the modulus values do not exceed 100 MPa, which also applies to materials added with CF fillers. The T_g_ recorded on the tan δ plots was almost constant for most of the tested samples (see Figure 12C), while the peak position ranged from 92 °C to 96 °C. The analysis of the tan δ plots revealed a lack of additional phase transition, which might suggest the presence of secondary immiscible polymer fractions. However, due to the similar T_g_ temperature range for PET, PETG, and PCTG resin, the results cannot exclude the existence of a two-phase structure of the PET/PETG and PET/PCTG blend.

That kind of behavior confirms that without heat treatment, the PET-based blends cannot achieve a sufficiently high content of the crystalline phase, which translates into exceptional sensitivity to temperature changes characteristic of amorphous polyester structures. Further heating of the samples confirms that the amorphous PET structures recrystallize at high temperatures, thereby strengthening the material. This is visible in the storage modulus graphs above 140 °C, where the modulus value increases visibly. The observed phenomenon of stiffness increase also took place during the annealing process at 150 °C. Unlike in the DMTA measurement conditions, the temperature in the furnace chamber was constant, and the procedure lasted 3 h, which allowed for full crystallization of the PET phase in the blends, but also avoided permanent deformation of the samples, which could be experienced by samples heated rapidly to higher temperatures. The resulting structures are characterized by much higher thermal resistance, since even at 100 °C, the modulus values reached at least 250 MPa, while for CF-reinforced materials, it can be even 1000 MPa. After reaching the glass transition region, the stiffness of the samples undergoes a visible decrease; however, unlike the untreated specimens (MEX-printed), the softening rate is less intensive. The consecutive heating leads to a further reduction in the storage modulus. Even at 150 °C, the modulus value for most of the materials did not drop below 100 MPa, which is a typical value for polyethylene at room temperature. The tan δ plots (see Figure 12D) confirm that the content of the amorphous phase was strongly reduced, since the peak area at the T_g_ region was significantly reduced. Additionally, the peak position for all samples was shifted to slightly higher temperatures, around 102–110 °C, which confirms that the increase in the PET crystalline phase content also influences the changes in the characteristics of macromolecules in amorphous areas. In this case, it is possible that the growing thickness of the PET phase lamellas influences the reduction in the mobility of PETG and PCTG chains, a significant part of which could be entangled in the interlamellar areas.

For the conducted study, the DMTA was supplemented with the thermomechanical test results analysis. For this purpose, the MEX-printed samples were examined using the heat deflection method (HDT) and Vicat softening (VST) technique. In both types of testing, we use two different load conditions, where the HDT measurements were conducted using 0.455 MPa and 1.8 MPa loads (see Figure 13), while for the VST method, 10 N and 50 N loads were applied (see Figure 14). Before the annealing procedure, the heat resistance for all samples was relatively low, which was also confirmed by the DMTA test results. However, considering that part of the sample was reinforced with a 10% of talc/CF filler, the obtained results are relatively low and range from 68 °C to 74 °C for the smaller load (0.455 MPa). For the higher load (1.8 MPa), the results are slightly lower and range from 65 °C to 71 °C. The differences recorded for particular samples are very small; however, the HDT for pure blends was recorded at the lowest temperature, while the addition of talc (T) and CF fillers improves the heat deflection temperature, which might be considered as an increasing trend. The differences between unmodified blends and composites are much more visible when the annealing procedure is applied, where the most significant were observed during small load HDT tests (0.455 MPa). The reference results for the rPET/PCTG/IM sample reached 112 °C, while the addition of the filler improved that value to 135 °C and 167 °C, respectively, for talc (T) and CF filler. The same measurements for the rPET/PETG/IM samples revealed the HDT of 108 °C for the unloaded blend, and 124 °C/174 °C when T/CF fillers were used. These results could be considered favorable for high-temperature applications; however, when results for a higher load (1.8 MPa) are considered, the measurement conditions strongly affect the values of thermomechanical parameters. The HDT values measured at higher load ranged from 75 to 77 °C for reference blends and talc-loaded specimens, while the values for CF-reinforced samples reached 82 °C and 93 °C, respectively, for the rPET/PCTG/IM-CF and rPET/PETG/IM-CF material. 

The Vicat test results for amorphous polyesters are usually within the glass transition temperature (T_g_) region, which is confirmed for the prepared materials. The result for the MEX-printed materials ranged from 72 °C to 86 °C, where, for a 10 N load, the VST values were slightly higher. Again, the heat treatment significantly improved the recorded Vicat temperatures. The same highest VST was measured for CF-reinforced materials, 169 °C and 194 °C, respectively, for rPET/PCTG/IM-CF and rPET/PETG/IM-CF samples. Equally high results were observed for the remaining samples, where the VST were in the range of 129–172 °C. The analysis of the graphs brings similar conclusions as in the case of HDT tests, where the results for the CF sample characterize these materials as more thermally resistant, which was also confirmed for Vicat tests conducted at a higher load of 50 N. The VST level was visibly reduced; however, the results for F reinforced samples still reached above 100 °C, 107 for the rPET/PCTG/IM-CF sample, and 122 °C for the rPET/PETG/IM-CF material. From a practical perspective, the improvement in HDT values can possibly be favorable in the medical application and food contact manufacturing processes. In both cases, there is a strong necessity for the preparation of personalized and/or customized components. For medical and biomedical applications, the possible use of heat-resistant materials is related to the preparation of patient-specific tools, gauges, and grippers. For most of the applications, all components need to be sterilized, sometimes at elevated temperatures around or above 100 °C. Similar high-temperature processes, like pasteurization or boiling, are used on the food processing lines. The 3D-printed parts/components can be used as spare parts or pilot line parts.

In summary, it is worth noting that the DMTA measurements confirmed that even above the temperature limit recorded during the HDT tests, the stiffness of the annealed material is still at a relatively high level, which means that the material can still successfully carry certain loads and does not quickly lose stability due to temperature changes, as is the case with materials after the MEX printing process. 

### 3.5. Rheological Characteristic—Small Amplitude Oscillatory Shear Measurements 

**Figure 15:**
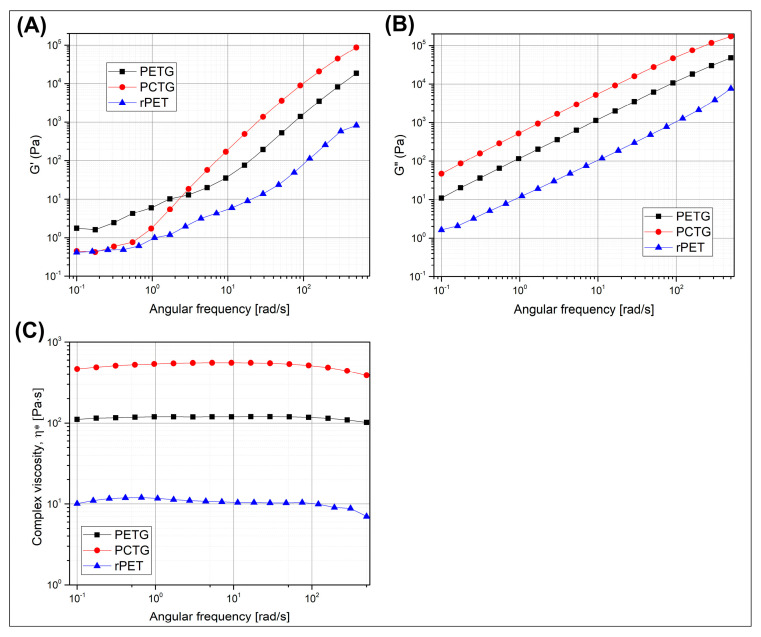
The rheological characteristics of the reference materials, rPET, PETG, and PCTG resin, are presented as (**A**) storage modulus–G″; (**B**) loss modulus–G″; (**C**) complex viscosity ƞ* plots.

**Figure 16:**
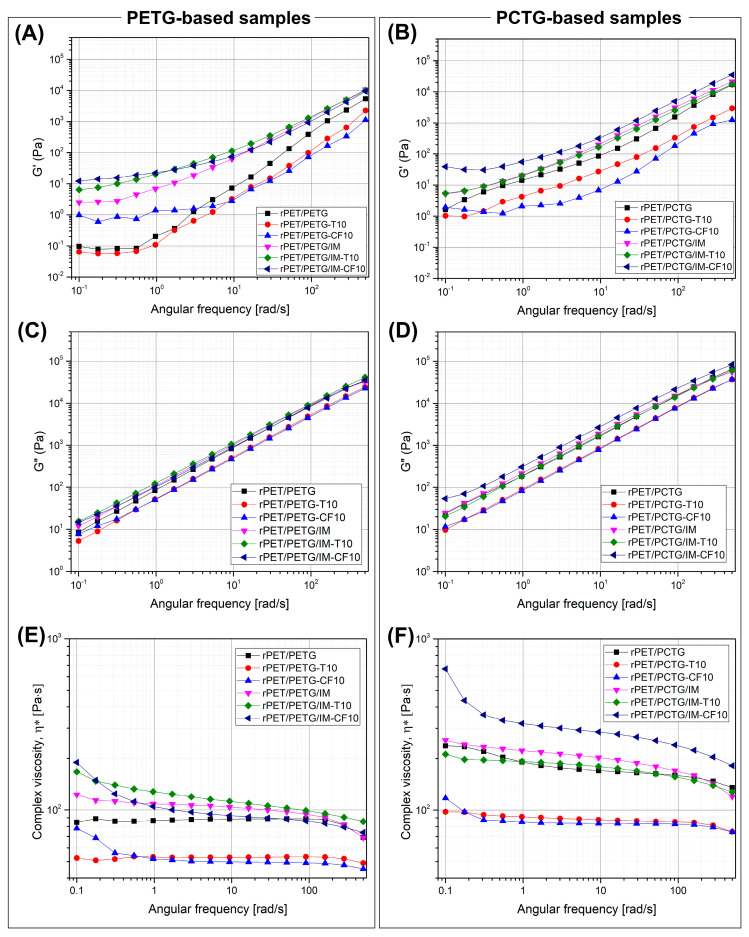
The rheological analysis results for the prepared blends: (**A**,**C**,**E**) rPET/PETG-based materials; (**B**,**D**,**F**) rPET/PCTG-based samples.

The rheological properties of the prepared samples were examined using a rotational rheometer, where the viscoelastic properties are presented in the form of storage/loss modulus plots (G′/G″) and complex viscosity results. The data are collected for the reference materials, rPET, PETG, and PCTG, see Figure 15, while for the prepared blends, the plots are presented in Figure 16. 

The results for the rPET resin are strongly influenced by the previously conducted processing stages. The supplied pellets were compounded from the recycled PET bottles; however, the quality of such post-consumer materials might be strongly influenced by the presence of other types of PET-based wastes, like flexible packaging, also prepared from the same virgin resin, but usually manufactured with the addition of sealing layers or paints. In addition, the level of macromolecular degradation for post-consumer materials also results from the repeated thermal loads to which the material is subjected. The sum of unfavorable factors usually leads to a reduction in the molecular weight and viscosity of materials, the rheological parameters of which often do not allow for effective processing of the packaging goods, not necessarily due to the reduction in viscosity, but mainly due to the instability of rheological parameters. The difference in the rheological characteristics is visible when comparing the reference samples. For the unmodified rPET resin, all viscoelastic properties are significantly different from those recorded for the copolymers used. Both G′ and G″ values are visibly lower than those of the virgin PETG/PCTG resin, while the viscosity difference is even more pronounced. For all three materials, the measurements were conducted in a nitrogen atmosphere, which limits the oxidative degradation; however, for all of the tested materials, the decomposition phenomenon was noticeable, since the viscosity in the low frequency range decreased visibly, which is a result of a relatively long test duration.

**Figure 17:**
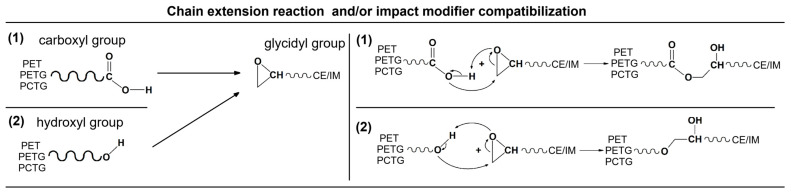
The reaction scheme presents the possible reactive extrusion products resulting from the carboxy and hydroxyl end groups’ reactions.

The rheological characteristics of the prepared blends need to be supplemented with additional comments. The prepared measurements are combining the results collected for materials from the preliminary compounding stage, where the rPET/copolymer (50%/50%) blends were compounded with the addition of talc (T) and CF. The main stage of material preparation was conducted with the use of elastomeric impact-modified (IM) and chain extender addition. The difference in rheological characteristics is therefore strongly influenced by the reactive processing conditions, since the glycidyl groups are present both in Joncryl type chain extender oligomer and POE-g-GMA elastomer [128,129,130,131]. Interestingly, the lowest complex viscosity, as well as G′/G″ results, were recorded for talc and CF blends from the preliminary stage. The samples were produced in the same procedure as mixtures without the addition of fillers; however, the characteristic for the unloaded blends revealed visibly higher viscosity, more similar to the results for samples from the second stage of tests, where reactive additives were already used. The possible reaction scheme for both the chain extender (CE) and the impact modifier additives is presented in Figure 17.

Assuming that for all blends the moisture level of the granulate after the drying procedure can be considered as practically similar, therefore, the potential reason for such differences in viscosity may be decomposition caused by the presence of moisture in fillers. In this case, the filler preparation process also included drying; however, it is possible that the standard drying conditions in the oven chamber are insufficient to completely remove water.

We did not analyze this phenomenon in detail because, ultimately, the problems with material degradation were solved by applying further modifications; however, taking into account the further potential for the development of this type of material, it can be pointed out that there is a need to improve the drying conditions for all input materials.

The direct comparison between the rPET/PETG and rPET/PCTG-based materials fully reflects the differences recorded for the reference materials, where PCTG-based materials are characterized by higher viscosity in the whole measured range. This type of result can be considered in two ways. For the current research, higher viscosity for the blend seems to be a slightly more favorable result, as it indicates improved flow characteristics compared to unmodified rPET, where even initial attempts to produce a filament proved very difficult due to insufficient melt strength of the extrudate. The addition of PCTG is visibly improving the viscosity, and the use of an additional reactive extrusion process with the presence of chain extender and elastomeric compound (IM) helps to improve the melt strength, especially for talc and CF-loaded materials, where the decomposition process in the preliminary study was more evident. On the other hand, in the case of potential applications of these types of materials in high-performance printing, where flow rates are significantly higher than for desktop machines, the increase in viscosity can be a negative phenomenon. In particular, it is worth paying attention to materials based on the PETG additive, where the filament extrusion process and the printing itself proceeded smoothly, despite the recorded low viscosities.

### 3.6. Printing Accuracy Evaluation—Complex Geometry Part 3D Scanning Results

**Figure 18:**
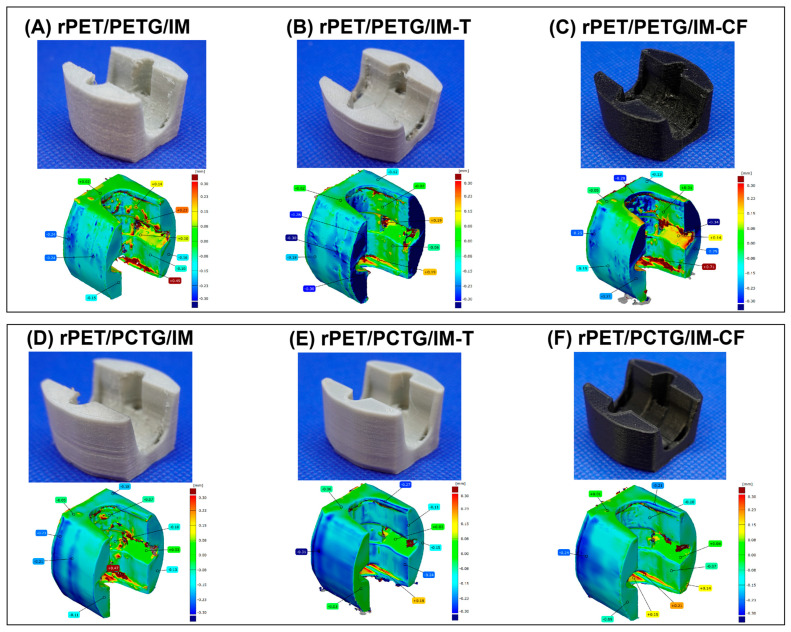
The results of the complex part printing tests for different types of materials. The general appearance of the prepared part was compared with the dimension deviations obtained from the surface 3d scanning. (**A**–**C**) The results for rPET/PETG/IM-based samples and (**D**–**F**) the analysis for the rPET/PCTG/IM samples.

The printing accuracy was measured based on a comparison of the STL model and a surface scan performed for several test parts prepared from the different types of rPET-based blends. The real appearance of the prepared model and the analysis of shape deviations with respect to the base 3D model are presented in Figure 18. The 3D model necessary to prepare test prints was downloaded from the Thingiverse portal (link: https://www.thingiverse.com/thing:24700, URL accessed on 30 January 2022).

Since the prepared part was designed as the holder for the linear bearing, it can be assumed that it is an example of a technical product requiring good mechanical properties. The shape of the chosen part is compact with numerous curves, several rectangular edges, and mounting holes. As a result, printing this type of component is relatively difficult despite its small size.

The part appearance presented in Figure 18 confirmed that, for the prepared materials, the warping problems were not observed. For all of the prepared samples, the lower surface has not undergone any deformation, and the side walls of the model do not show any defects such as delamination or separation of the model layers, which could suggest excessive shrinkage or lack of cohesion of the layers. Unfortunately, during the test trials, frequent problems with the stringing of the material were observed, which led to the appearance of burrs on the surface of the prints. These instabilities are also leading to large defects in the area of the seam line. For the prepared models, most of these types of defects appear on the inner walls, while the outer surfaces usually do not show these types of defects. However, this does not change the fact that such defects may cause failures during the operation of the finished component, caused by the poor fit of the bearing element with the mounting.

3D scanning results and deviation measurements can be interpreted for flat external surfaces; any attempts to dimension internal surfaces are subject to significant inaccuracy caused by surface defects. Despite the relatively similar absolute deviation values between the rPET/PETG and rPET/PCTG samples, the surface of the PCTG-based sample is more uniform, while the PETG-based materials show a slightly greater tendency to form irregular lateral surfaces. Considering the rheological differences, with the rPET/PCTG samples exhibiting higher viscosity, this may be a likely cause of these defects. The dimensional deviation for any of the significant side surfaces of the prepared parts did not exceed 0.3 mm, while most scans suggest a deviation range of ±0.15 mm in relation to the original model. This suggests a lack of significant problems with model deformation caused by excessive shrinkage or delamination of the material structure. Unfortunately, most of the visible defects are caused by quite intense stringing, which is a significant problem, especially for complex geometric models. However, it is worth noting that for most materials, such problems can be eliminated by appropriately optimizing the printing parameters.

## 4. Conclusions

The results of the presented research work can be summarized as a broad comparative study of the developed composite materials. Due to several thematic areas, the conclusions can be divided into several main sub-points, each of which can be used to assess individual aspects of the presented research concept.

- The use of the rPET seems to be a valuable component for the preparation of filament materials. However, it is worth noting that for a particular type of recycled resin, the original properties were strongly deteriorated during the recycling procedure. Reduction in molecular weight was influencing the rheological characteristics, which limits the melt strength. The mechanical properties of the rPET samples were also influenced by brittleness. The melt blending with the PETG/PCTG copolymers is not leading to deterioration of properties; the mixture is characterized by miscibility or at least good compatibility.
- The addition of fillers to the prepared blend system confirms the favorable properties of the CF component, while for talc (T) particles, it was visible that the reinforcing efficiency at such a low content (10% wt.) was not sufficient to improve the mechanical properties.
- The annealing procedure can be considered as an effective method for enhancing the heat resistance of the PET-based blends; however, considering the direct comparison with pure PET, there is some room for improvement.
- The use of an elastomeric impact modifier (IM) was crucial for the successful fabrication of the filament samples. The brittleness is still an important factor limiting the development of PET-based materials; however, the results of the toughness parameters (elongation, impact strength) revealed that the use of PCTG component was more favorable than the PETG resin, which is, in our opinion, a significant advantage of this relatively less popular material.

The conducted studies can be treated as the reference point for further studies, where suggested modifications can be implemented as part of the next study. The most probable directions of research include the use of larger amounts of polymer additives, while in the case of research on the polymer matrix, an interesting direction of work may be further optimization of the mixture composition towards improving the impact strength.

## Figures and Tables

**Table 1:** The sample marking and materials formulations.

	rPET	PETG	PCTG	IM (Impact Modifier)	CE (Chain Extender)	Filler (T/CF)
	[wt.%]	[wt.%]	[wt.%]	[wt.%]	[wt.%]	[wt.%]
Preliminary study						
rPET/PETG	50	50	- *	-	-	-
rPET/PETG-T	45	45	-	-	-	T 10%
rPET/PETG-CF	45	45	-	-	-	CF 10%
rPET/PCTG	50	-	50	-	-	-
rPET/PCTG-T	45	-	45	-	-	T 10%
rPET/PCTG-CF	45	-	45	-	-	CF 10%
Impact-modified materials						
rPET/PETG/IM	40	40	-	19.5	0.5	-
rPET/PETG/IM-T	36	36	-	17.55	0.45	T 10%
rPET/PETG/IM-CF	36	36	-	17.55	0.45	CF 10%
rPET/PCTG/IM	40	-	40	19.5	0.5	-
rPET/PCTG/IM-T	36	-	36	17.55	0.45	T 10%
rPET/PCTG/IM-CF	36	-	36	17.55	0.45	CF 10%

* the hyphen mark “-” means that the particular compound was not used

**Table 2:** The data collected from the DSC analysis.

	Peak Position [°C]			Enthalpy [J/g]		Crystallinity [%]
Sample	T_m_	T_cc_	T_c_	ΔH_cc_	ΔH_m_	X_c_
Preliminary stage materials						
rPET	253.6	130.0	194.1	23.36	41.5	12.9
rPET/PETG	247.6	138.6	-	17.85	36.24	26.3
rPET/PETG-T	244.9	134.3	172.3	22.29	34.28	19.0
rPET/PETG-CF	246.6	138.0	154.0	26.57	37.36	17.1
rPET/PCTG	251.3	147.7	-	6.145	27.56	30.5
rPET/PCTG-T	251.5	127.8	167.3	15.14	28.94	21.9
rPET/PCTG-CF	250.9	137.6	148.1	20.85	33.14	19.5
Impact-modified materials						
rPET/PETG/IM	247.1	139.4	151.0	20.87	30.79	17.7
rPET/PETG/IM-T	247.6	136.2	166.2	20.1	30.21	20.0
rPET/PETG/IM-CF	246.3	135.3	151.7	21.23	29.37	16.1
rPET/PCTG/IM	251.6	131.0	149.2	12.11	27.07	26.7
rPET/PCTG/IM-T	251.3	129.8	157.9	12.15	23.86	23.2
rPET/PCTG/IM-CF	251.6	129.0	147.1	14.89	25.68	21.4
Annealed samples						
rPET/PETG/IM	246.5	-	171.8	-	30.83	55.0
rPET/PETG/IM-T	243.1	-	156.9	-	35.1	69.6
rPET/PETG/IM-CF	245.0	-	159.6	-	32.02	63.5
rPET/PCTG/IM	249.2	-	145.9	-	28.21	50.3
rPET/PCTG/IM-T	250.0	-	162.9	-	28.21	55.9
rPET/PCTG/IM-CF	246.8	-	147.1	-	26.27	52.1

## Data Availability

The original contributions presented in this study are included in the article. Further inquiries can be directed to the corresponding author.

## Supplementary Materials

The following supporting information can be downloaded at: https://www.mdpi.com/article/10.3390/polym18060768/s1, Figure S1: The results of the DSC analysis for the reference materials (rPET, PCTG, PETG): (A) 1st heating; (B) cooling; (C) 2nd heating; Table S1: Mechanical properties for all of the prepared materials.
