# Supplementary material for: The Use of Recycled Poly(Ethylene Terephthalate)/Amorphous Polyester Blends/Composites in Materials Extrusion (MEX) Additive Manufacturing Techniques: The Influence of Talc and Carbon Fiber on the Mechanical Performance and Hear Resistance
Source: Polymers (Basel). 2026 Mar 22;18(6):768. doi: 10.3390/polym18060768 (PMC13030397; doi:10.3390/polym18060768)
Supplement: Supplementary file 1 [file polymers-18-00768-s001.zip › polymers-4205126-supplementary.pdf]

Supplementary Information Section

The use of recycled poly(ethylene terephthalate)/amorphous polyester blends/composites in materials extrusion (MEX) additive manufacturing techniques: the influence of talc and carbon fiber on the mechanical performance and hear resistance

Abstract

The conducted study was focused on the development of the new type of polymer blends intended for additive manufacturing applications, in particular the material extrusion method (MEX). The developed materials were prepared from recycled poly(ethylene terephthalate) and amorphous copolymers poly(ethylene terephthalate-glycol) (PETG), and poly(cyclohexylenedimethyl terephthalate-glycol) (PCTG). The basic blend systems were additionally modified with POE-g-GMA impact modifier (IM) during the reactive extrusion process. The main aim of the work was to assess the effectiveness of using composite additives and their influence on the mechanical and thermomechanical parameters of the tested systems. To prepare the composites, selected polymer blends were modified with 10% of talc (T) and carbon fibers (CF). The properties evaluation include the mechanical/thermomechanical testing, thermal analysis and structural observations. The accuracy of printing was measured using optical scanning methods. The test results indicate that even the relatively small amount of the CF filler could lead to a significant increase in tensile modulus from reference 1.6 GPa to 2.9 GPa, the same improvement applies to strength values, where the CF-modified materials reached 45 MPa, compared to the reference 31 MPa. The heat deflection tests (0.455 MPa) after annealing revealed the maximum HDT of around 170 °C for both types of CF-modified materials. The Vicat test results were also favorable for annealed materials. Considering that the Vicat/HDT results after the 3D printing process usually reach around 70 °C, the performed heat treatment strongly enhanced the heat resistance for most of the prepared blends. The performed studies revealed that for most of the prepared materials, the brittleness was a common drawback for both MEX-printed and injection molded materials.

Keywords: polymer blends; poly(ethylene terephthalate); extrusion 3D printing (MEX); recycling; polymer composite; thermal properties; talc; carbon fibers

Table S1. Mechanical properties for all of the prepared materials

Sample	Tensile strength [MPa]	Tensile modulus [MPa]	Elongation at break [%]	Charpy impact strength [kJ/m^2]
**Injection molded samples**				
PCTG	45.4 (±0.1)	1870 (±5)	160 (±28)	11.6 (±3.9)
PETG	55.0 (±0.6)	2350 (±13)	17 (±6.4)	2.9 (±0.3)
rPET	61.9 (±0.2)	2640 (±16)	130 (±37)	3.1 (±0.5)
rPET/PETG/IM	36.7 (±0.3)	1500 (±15)	18 (±5.6)	5.9 (±0.6)
rPET/PETG/IM-T	34.9 (±0.8)	1720 (±17)	4.1 (±0.2)	2.9 (±0.3)
rPET/PETG/IM-CF	55.6 (±0.4)	3170 (±67)	5.3 (±0.7)	7.6 (±0.2)
rPET/PCTG/IM	33.0 (±0.8)	1440 (±20)	37 (±9.4)	10.2 (±0.6)
rPET/PCTG/IM-T	33.6 (±0.6)	1600 (±16)	5.3 (±0.3)	3.7 (±1.1)
rPET/PCTG/IM-CF	61.4 (±4.7)	3450 (±324)	7.5 (±0.7)	8.8 (±0.6)
**Injection molding/annealing**				
rPET/PETG/IM	37.1 (±2.3)	1640 (±105)	3.0 (±0.2)	2.1 (±0.5)
rPET/PETG/IM-T	30.5 (±0.7)	1910 (±8)	2.2 (±0.1)	1.3 (±0.2)
rPET/PETG/IM-CF	66.4 (±6.6)	3370 (±420)	3.4 (±0.5)	2.6 (±0.1)
rPET/PCTG/IM	36.6 (±0.3)	1590 (±28)	33 (±5.1)	3.6 (±1.0)
rPET/PCTG/IM-T	36.1 (±1.2)	1730 (±50)	3.5 (±0.5)	1.8 (±0.1)
rPET/PCTG/IM-CF	55.5 (±3.8)	2600 (±159)	4.6 (±1.4)	5.3 (±0.2)

3D printed samples				
rPET/PETG/IM	31.3 (±1.1)	1620 (±32)	4.5 (±0.9)	3.2 (±0.9)
rPET/PETG/IM-T	31.9 (±0.2)	1930 (±50)	3.4 (±0.2)	2.9 (±0.5)
rPET/PETG/IM-CF	45.1 (±0.2)	2920 (±134)	4.6 (±0.1)	6.1 (±0.5)
rPET/PCTG/IM	33.2 (±0.5)	1520 (±28)	5.3 (±0.5)	1.1 (±0.1)
rPET/PCTG/IM-T	28.4 (±1.8)	1600 (±60)	3.4 (±0.2)	3.5 (±0.5)
rPET/PCTG/IM-CF	39.7 (±0.7)	2730 (±57)	3.3 (±0.1)	7.2 (±0.8)
3D Printing/annealing				
rPET/PETG/IM	23.5 (±4.0)	1340 (±76)	1.9 (±0.3)	1.0 (±0.1)
rPET/PETG/IM-T	19.2 (±3.4)	1390 (±134)	1.5 (±0.2)	0.9 (±0.1)
rPET/PETG/IM-CF	29 (±0.7)	2540 (±146)	2.8 (±0.1)	2.2 (±0.1)
rPET/PCTG/IM	18.4 (±0.1)	1670 (±69)	1.1 (±0.1)	0.8 (±0.2)
rPET/PCTG/IM-T	19.3 (±1.6)	2050 (±35)	1.0 (±0.1)	1.4 (±0.4)
rPET/PCTG/IM-CF	30.7 (±1.2)	3280 (±72)	1.8 (±0.1)	3.2 (±0.3)

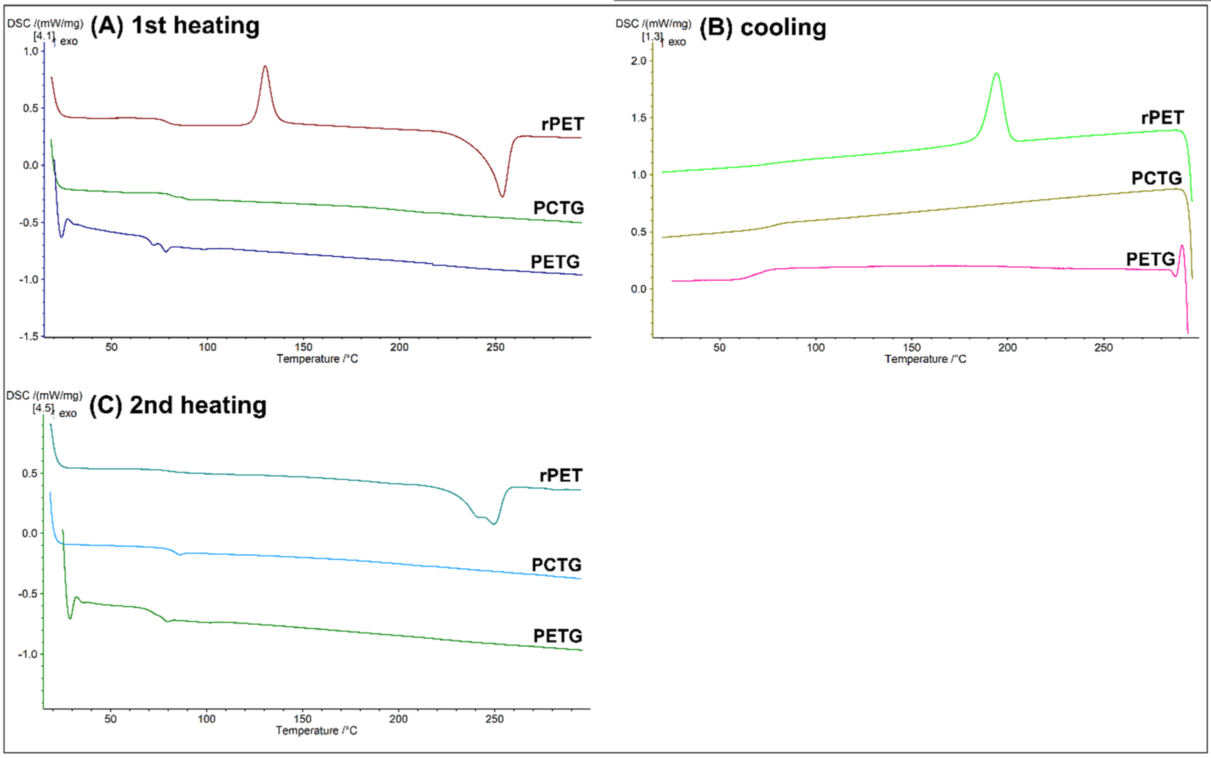

Figure S1. The results of the DSC analysis for the reference materials (rPET, PCTG, PETG): (A) 1st heating; (B) cooling; (C) 2nd heating.
